# Digitally Fabricated and Naturally Augmented In Vitro Tissues

**DOI:** 10.1002/adhm.202001253

**Published:** 2020-11-16

**Authors:** Daniela F. Duarte Campos, Laura De Laporte

**Affiliations:** ^1^ Department of Advanced Materials for Biomedicine Institute of Applied Medical Engineering RWTH Aachen University Aachen 52074 Germany; ^2^ DWI—Leibniz Institute for Interactive Materials Aachen 52074 Germany; ^3^ Department of Technical and Macromolecular Chemistry RWTH Aachen University Aachen 52074 Germany

**Keywords:** bioprinting, computational modeling, digital fabrication, hydrogels, in vitro tissues

## Abstract

Human in vitro tissues are extracorporeal 3D cultures of human cells embedded in biomaterials, commonly hydrogels, which recapitulate the heterogeneous, multiscale, and architectural environment of the human body. Contemporary strategies used in 3D tissue and organ engineering integrate the use of automated digital manufacturing methods, such as 3D printing, bioprinting, and biofabrication. Human tissues and organs, and their intra‐ and interphysiological interplay, are particularly intricate. For this reason, attentiveness is rising to intersect materials science, medicine, and biology with arts and informatics. This report presents advances in computational modeling of bioink polymerization and its compatibility with bioprinting, the use of digital design and fabrication in the development of fluidic culture devices, and the employment of generative algorithms for modeling the natural and biological augmentation of in vitro tissues. As a future direction, the use of serially linked in vitro tissues as human body‐mimicking systems and their application in drug pharmacokinetics and metabolism, disease modeling, and diagnostics are discussed.

## Graphical abstract

Intertwining computational design and modeling for tissue engineering using manufacturing technologies like bioprinting is a topic of rising interest. This progress report discusses advances of bioink polymerization simulation, design of fluidic devices for the culture of in vitro tissues, and the use of computational models for the simulation of in vitro tissue natural augmentation.

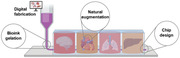

## 1 Introduction

Animal models are a cornerstone of biomedical research. Yet they are expensive, ethically polemic, and limited in understanding the prognosis of human disease.^[^ ^1^ ^]^ In particular, it is challenging to sufficiently address phenotypic, genotypic, and physiological differences between animals and humans. Despite these shortcomings, there are currently few alternatives to animal experiments for the study of most diseases and testing of new drugs. Human tissue explants are scarce and not compatible with long‐term experiments. For these reasons, more sophisticated, human‐engineered in vitro tissues and fluidic models are interesting complementing systems to animal models, which can offer higher controllability, modularity, reproducibility, and include scale‐up advantages.

**Figure 1:**
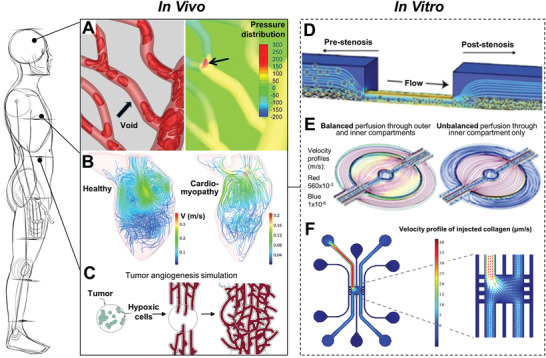
Overview of computer‐aided design and computational fluidic dynamics used in biomedical research. Computed models are important tools in simulating both healthy and pathologic conditions in vivo and in vitro. Computational modeling of A) blood flow in vivo,^[^ ^11^ ^]^ B) hypertrophic cardiomyopathy in vivo,^[^ ^12^ ^]^ C) tumor‐triggered angiogenesis in vivo,^[^ ^13^ ^]^ D) stenosis in vitro,^[^ ^15^ ^]^ E) in vitro diabetes model with a compartmentalized adipocyte‐immune cell coculture,^[^ ^16^ ^]^ and F) cancer transendothelial migration model in vitro.^[^ ^17^ ^]^ Images reproduced and adapted with permission: (A)^[^ ^11^ ^]^ Copyright 2017, Elsevier; (B)^[^ ^12^ ^]^ Copyright 2016, the Authors. Published by Springer Nature; (C)^[^ ^13^ ^]^ Copyright 2004, Royal Society; (D)^[^ ^15^ ^]^ Copyright 2012, American Association for the Advancement of Science (AAAS); (E)^[^ ^16^ ^]^ Copyright 2001, Royal Society; (F)^[^ ^17^ ^]^ Copyright 2018, the Authors. Published by Springer Nature.

Since the end of the 20th century, there is a trend for materials scientists, biologists, and physicians to create lab‐grown, engineered in vitro tissues.^[^ ^2^, ^3^, ^4^ ^]^ Recently, attention is rising to intersect materials and natural sciences with arts and informatics, such as digital design and computational modeling.^[^ ^5^, ^6^, ^7^, ^8^, ^9^ ^]^ Computer‐aided design and fluidic simulations have been used for decades in clinical imaging to model, for example, blood flow in vivo.^[^ ^10^ ^]^ Vascular networks are responsible for the transport of nutrients and other substances to tissues (**Figure** **1**A).^[^ ^11^ ^]^ These functions can be hindered when pathological conditions ensue. For example, hypertrophic cardiomyopathy is a myocardial defect that is characterized by an excessive thickening of the left ventricle myocardium and can cause sudden heart failure (Figure 1B).^[^ ^12^ ^]^ Therefore, computational fluidic dynamics (CFD) simulation of this medical condition can provide useful insights to understand the intraventricular blood flow dynamics. In cancer research, the recruitment of new blood vessels, i.e., angiogenesis by cancerous tumors can be modeled using growth numerical simulation (Figure 1C).^[^ ^13^ ^]^ With the pressure to reduce the use of animal models, the demand for in vitro systems that recapitulate these in vivo settings is increasing.

For cardiovascular disease, several microfluidic platforms have been used for in vitro drug screening against thrombosis and stenosis.^[^ ^14^ ^]^ Poly(lactic‐*co*‐glycolic acid) (PLGA) nanoparticles coated with a clot‐dissolving drug (tissue plasminogen activator) were used as a mechanically activated drug delivery system to dissolve blood clots under high shear stress. Aggregates of thrombolytic nanoparticles in the micrometer size broke apart when exposed to mechanical forces. First, the effect of drugs on stenosis under different shear rates was investigated with CFD simulations, which predicted that low shear rates led to longer occlusion times, while at increased shear rates, naturally occurring at the obstruction site, drugs better alleviated blood clots (Figure 1D).^[^ ^15^ ^]^ In silico physiological inlet shear rates of about 1000 s^−1^ led to pathological levels of about 100 000 s^−1^ at 90% lumen occlusion. After simulation, the efficacy of drug‐loaded nanoparticles was validated in a mouse pulmonary embolism model, which showed that upon separation of nanoparticle aggregates, the pulmonary artery pressure was normalized within 1 h.

Alongside cardiovascular diseases, diabetes is also highly prevalent among adults worldwide. Therefore, fluidic models were developed to better understand the crosstalk between immune cells and adipocytes, which is commonly associated with chronic inflammation in obese individuals.^[^ ^16^ ^]^ In these models, a constant rate of nutrient supply and waste removal was tested in a compartmentalized silicone microfluidic coculture system, where adipocytes and immune cells were cocultured in different compartments separated by a porous barrier. To mimic conditions with varying nutrient supply, the inner compartment with adipocytes and the outer compartment with immune cells were perfused differently with cell‐specific medium, while finite element analysis quantified the varying velocity (Figure 1E). When the medium was perfused only through the inner compartment, the velocity profiles in the outer compartment were extremely low, thus, limiting nutrient supply for the immune cells (Figure 1E, right). When the medium was perfused through both compartments, there was a balanced supply of both cell‐specific media (Figure 1E, left), improving adipocyte differentiation, which was required to build up the in vitro diabetic model. Using the latter experimental setup, insulin‐stimulated adipocytes that were cocultured with immune cells showed a moderate increase (1.4‐fold) in glucose uptake, thus, indicating a tendency for insulin resistance in comparison with adipocytes cultured in single cultures without immune cells. Models such as this can potentially also be applied in the future to study other obesity‐related diseases, besides diabetes.

In cancer research, numerical simulation was employed to develop a microfluidic system that can replicate cancer cell transmigration from a vessel lumen to the surrounding extracellular matrix (ECM) by allowing for live‐cell imaging and analysis of the culture chambers. The ECM was represented in vitro by a collagen gel contained in a polydimethylsiloxane (PDMS) microchannel, while blood vessels were reconstructed in its vicinity with direct contact. Collagen gel was injected into 100 µm microchannels and the flow velocity profile of the collagen gel (2 mg mL^−1^, 6 mPa s viscosity) during injection was simulated to ensure complete filling of the microchannels (Figure 1F).^[^ ^17^ ^]^ The confinement of the collagen gel in the central region of the fluidic system strongly relied on the channel geometry, thus highlighting the need for specific device design before in vitro testing. After in silico tests, collagen gels were injected into the chips with an inlet velocity of 50 µm s^−1^. For microvessel formation, type I and IV collagen were added to the smaller channels contained in the PDMS device to coat inside of the lumen. Excess of unbound collagen was washed away with medium and human umbilical vein endothelial cells (HUVECs) were seeded for lumen endothelialization. The formed microvessel‐like lumen showed a permeability of about 12 × 10^−6^ cm s^−1^ to 70 kDa dextran, which is biologically acceptable, in comparison with other literature reported systems. When cancer cells were flowing inside the vessels, HUVECs acted as a regulated barrier to prevent uncontrolled transendothelial migration.

Bioprinting is a key biofabrication method to create artificial in vitro tissues and organs by the sequential deposition of cell‐laden bioink layers.^[^ ^18^, ^19^ ^]^ So far, interesting examples have demonstrated the promise of bioprinting to create in vitro tissues and disease models. For instance, microextrusion bioprinting was used to generate extension lattices for neural research,^[^ ^20^ ^]^ laser‐based bioprinting to construct 3D coculture models of interacting cancer and endothelial cells (ECs),^[^ ^21^ ^]^ and drop‐on‐demand bioprinting to recreate the neural stem cell niche in 3D.^[^ ^22^ ^]^ Therefore, it is advantageous to combine bioprinting, which helps capturing the 3D heterogeneity of native tissues, with fluidic culture devices with integrated vascularization that is necessary for tissue growth, maturation, and biological augmentation.^[^ ^22^, ^23^, ^24^ ^]^ For this reason, advances in computational modeling used in tissue engineering, digital design, and fabrication used in the development of fluidic culture devices, and generative algorithms employed for modeling the growth of in vitro tissues are exciting aspects, discussed in the following sections.

## 2 Computational Modeling of Hydrogels to Engineer In Vitro Tissues

Human tissues consist of three key components: cells, ECM, and soluble factors. The development of artificial tissues and organs in the lab has evolved from 2D to 3D cultures over the past two decades. Natural and synthetic hydrogels play a leading role in tissue engineering as supporting matrices that mimic the native ECM.^[^ ^2^, ^25^, ^26^ ^]^ In particular, hydrogel precursor solutions are a major component of so‐called bioinks and are mixed with cells. A major challenge remains that their properties have to be compatible with bioprinting.^[^ ^27^, ^28^, ^29^ ^]^ Bioink polymerization methods, gelation kinetics, and rheological properties can influence the success of the printing process, 3D geometrical fidelity, and biological outcomes, such as cell survival, phenotype maintenance, and differentiation potential.^[^ ^30^, ^31^, ^32^, ^33^ ^]^ In this section, we discuss how computational modeling can help predicting bioink polymerization kinetics before, during, and after printing, and why bioink rheological properties should be modeled and designed to fit different bioprinting methods to minimize their impact on cell viability.

### 2.1 Modeling Physical and Chemical Bioink Polymerization

Bioink polymerization can be categorized as physical, for example by thermal crosslinking (temperature change) and ionic crosslinking (presence of ions), or chemical, such as photocrosslinking (UV light).^[^ ^25^ ^]^ Agarose, gelatin, collagen, and silk fibroin are examples of thermo‐responsive hydrogels with physical polymerization, whereas alginate, chitosan, and nanocellulose have an ionic crosslinking mechanism. Fibrin, polyethylene glycol (PEG), and polyglycidol (PG) undergo irreversible chemical crosslinking, while the crosslinks can be broken by the presence of domains sensitive to hydrolysis or proteolysis.^[^ ^34^ ^]^ Depending on the reactive endgroups of PEG and PG, crosslinking can be light‐induced (currently mainly UV) via free‐radical polymerization or thiol‐ene click chemistry. Bioink polymerization kinetics are governed by the crosslinking type and rheological properties.^[^ ^35^ ^]^ For instance, low viscosity collagen hydrogels (type I, 1.25 mg mL^−1^) have slower polymerization kinetics compared to fibrin (12.5 mg mL^−1^ fibrinogen, 20 U mL^−1^ thrombin), alginate (1% w/v), and PEG diacrylate (PEGDA, MW 3400, 15 wt%, 0.05% w/v Irgacure).^[^ ^36^, ^37^ ^]^

When designing hydrogels for 3D cell and tissue growth, there is a complex interplay between the stiffness, viscoelasticity, mesh size, degradation rate, and cell ligand density. How these individual parameters affect cell behavior is still difficult to analyze and decouple. For example, hydrogels formed via free‐radical polymerization (e.g. gelatin methacrylate, GelMA) or via click‐reactions (e.g. thiol‐ene, Michael‐type) will lead to very different internal structures.^[^ ^34^, ^38^ ^]^ Especially uncontrolled free‐radical crosslinking leads to polymer‐network gels with nano‐ to microstructural spatial inhomogeneity in terms of polymer‐segments and crosslinking densities.^[^ ^39^ ^]^ Also, the gelation kinetics can influence the hydrogel network and the resolution of the macroscopic architecture. On the other hand, natural materials like proteins^[^ ^40^ ^]^ or self‐assembling peptides,^[^ ^41^ ^]^ or hybrid molecules like polyisocyanides^[^ ^42^ ^]^ can form more fibrous, viscoelastic, and strain stiffening structures, which better mimic the ECM. However, in the case of natural materials, it remains challenging to control the biochemical, mechanical, and physical parameters, which are also difficult to decouple and may vary from batch to batch. Therefore, to better predict cell behavior in bioprinted constructs, physico‐chemical models are required to predict the spatiotemporal forces cells are experiencing due to the hydrogel properties, which will determine their migration, proliferation, and in the case of stem cells, differentiation. To link simulation with experiments, new methods are required to analyze local network structures and its degradation dynamics. The material mesoscopic morphology (mesh size and distribution in the range of ≈1 nm to few µm) can be investigated with light, small‐angle X‐ray (SAXS), and neutron scattering (SANS) and fitted to structural models.^[^ ^43^ ^]^ The scattering data can reveal spatial correlations between the polymer strands of the network and cross‐linkers from complex hydrogels. Based on the type of crosslinks, the printed materials can be adaptive to their cellular environment or due to transient connectivity in response to mechanical forces or external triggers. Therefore, more understanding is necessary of the mutual interplay between biomaterial (nano)structure, dynamics, and properties and how they can be engineered to direct cells and tissue maturation.^[^ ^44^ ^]^

**Figure 2:**
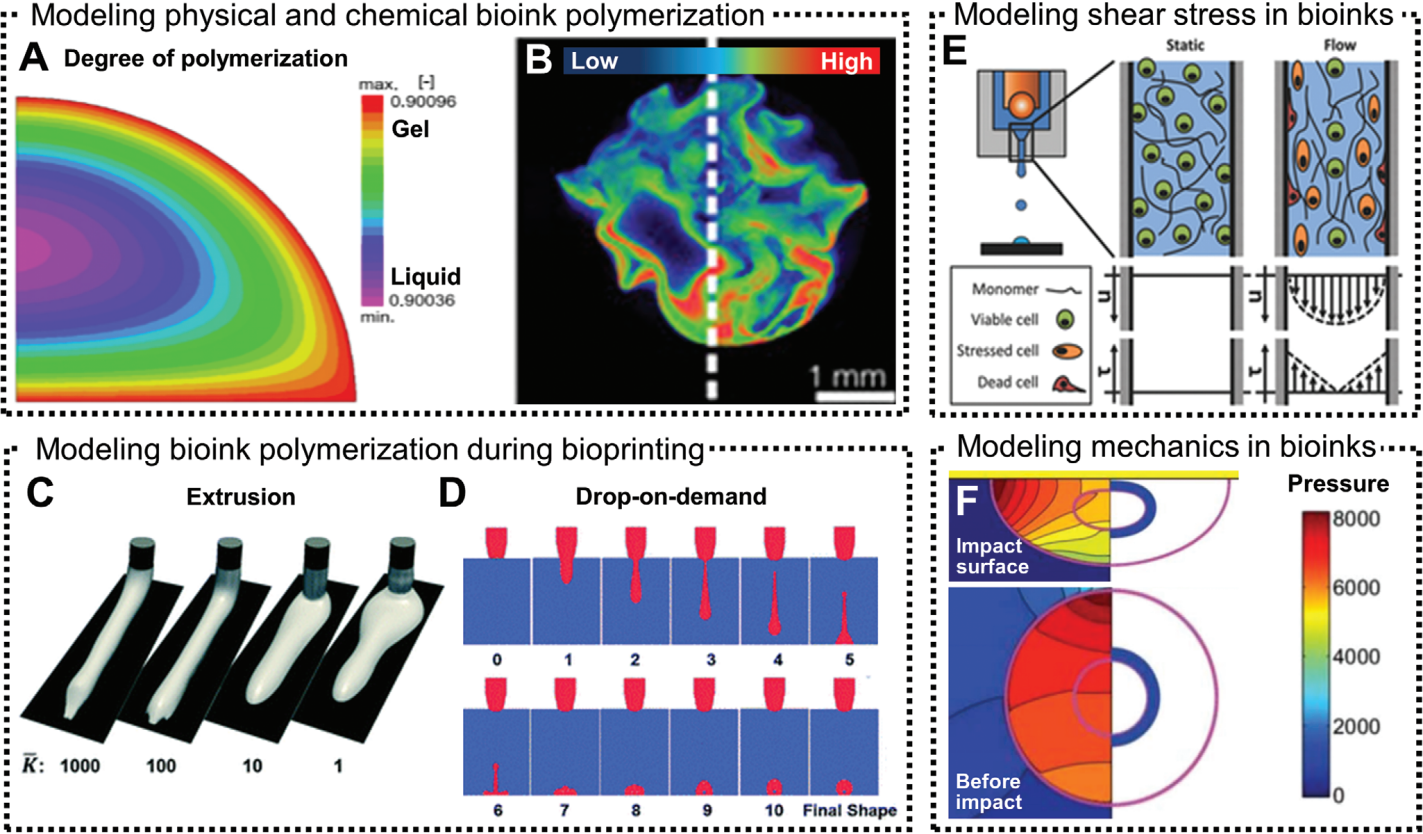
Numerical models used to simulate bioink gelation kinetics and printing performance. A) Finite element analysis of agarose droplet gelation kinetics.^[^ ^45^ ^]^ The computed degree of polymerization is unitless and is displayed in color as liquid state (purple/blue) and gel state (red, yellow). B) Experimental demonstration of heterogeneous crosslinking of hydrogels with Michael‐type addition crosslinking mechanism showing regions of low (blue) and high (red) degree of polymerization.^[^ ^46^ ^]^ C) Numerical modeling of bioink's viscosity and extrusion speed on the printing performance.^[^ ^54^ ^]^ D) Simulation of droplet formation during drop‐on‐demand bioprinting.^[^ ^54^ ^]^ E) Shear stress profiles in static and flow conditions during drop‐on‐demand bioprinting and its impact on cell viability.^[^ ^30^ ^]^ F) Computational modeling of a droplet's impact on a flat surface, before the impact (lower image) and after impact (upper image).^[^ ^59^ ^]^ Pressure distribution on the cell body is displayed in color as high (red) and low (blue). Images reproduced and adapted with permission: (A)^[^ ^45^ ^]^ Copyright 2016, John Wiley and Sons; (B)^[^ ^46^ ^]^ Copyright 2016, Elsevier; (C,D)^[^ ^54^ ^]^ Copyright 2001, Royal Society; (E)^[^ ^30^ ^]^ Copyright 2015, Wiley‐VCH GmbH; (F)^[^ ^59^ ^]^ Copyright 2018, Elsevier.

In bioprinting, one‐ or two‐component printing strategies can be applied depending on the bioink crosslinking type. One component bioinks with thermal or photo‐crosslinking can be printed using a single printer head that either extrudes the bioink or ejects small droplets onto the printing substrate. Bioinks with ionic or enzymatic crosslinking require either a two‐component coaxial printing nozzle that mixes both components during printing or a single nozzle that prints the bioink inside a bath containing the crosslinking solution. The gelation process of one‐component printing of thermo‐reversible agarose hydrogels has been modeled by finite element analysis (**Figure** **2**A).^[^ ^45^ ^]^ This in silico model showed that the physical gelation of single agarose droplets during drop‐on‐demand printing is temperature‐, concentration‐, and time‐dependent. From a practical perspective, this outcome can be utilized to redesign the printer head and nozzle with temperature‐controllable elements that help speeding up or slowing down the polymerization kinetics of the bioink during the printing process, so that uniform spherical droplets are formed.

Simulating the gelation of bioinks with ionic, enzymatic, click, or free‐radical crosslinking is more challenging than the example mentioned above, as they require two components for polymerization or rely on an initiation reaction, respectively. Even though fast gelation kinetics are desired to achieve high printing resolutions, synthetic hydrogels with very rapid gelation kinetics, for example via the thiol‐maleimide Michael‐type reaction, have shown inefficient mixing, heterogeneous gelation, and inconsistent cell responses.^[^ ^46^ ^]^ Importantly, the printing of two‐component hydrogel precursor solutions requires efficient mixing strategies without clogging the nozzle. In an alternative approach, a printing head with two piezoelectric pipettes was employed to eject and fuse two droplets to enable simultaneous dispensing and crosslinking of two components before and upon contact with the substrate.^[^ ^47^ ^]^ To render two‐component bioinks compatible with bioprinting and tissue engineering, it is desirable to simulate their gelation kinetics and analyze their microstructure beyond visual inspection. Although the gelation kinetics of hydrogels with Michael‐type addition crosslinking mechanisms have not been computationally simulated in this context yet, it has been experimentally shown that by systematically designing the di‐thiol‐containing matrix metalloproteinase (MMP) sensitive peptide crosslinker with a tag sequence (XCXX) improved maleimide or vinylsulfone thiolate reaction (Figure 2B).^[^ ^46^, ^48^ ^]^ In short, these studies highlight the fact that using in silico gelation methods before experimental testing is beneficial to select the best hydrogel candidates as bioinks and design the nozzles accordingly for a given bioprinting method and biomedical application.

### 2.2 Modeling Bioink Polymerization before, during, and after Fabrication

Current bioprinting strategies using a printing nozzle can be categorized into extrusion‐ and droplet‐based.^[^ ^18^ ^]^ Each printing method is compatible with one or more types of bioinks that crosslink in distinct ways. Extrusion bioprinting is by far the most frequently used bioprinting method as it is compatible with a wide range of injectable hydrogels developed for regenerative medicine applications.^[^ ^28^, ^49^, ^50^, ^51^, ^52^, ^53^ ^]^ Briefly, it consists of extruding thin strands of pre‐polymerized bioinks or uncrosslinked bioink precursors through a needle onto a printing platform or into a bath containing a crosslinking solution by applying pressurized air to the printer head. This procedure can also be done using more than one printer head and is repeated layer‐by‐layer until a 3D construct is generated. In a recent study, researchers used numerical modeling to simulate the effect of bioink viscosity and extrusion rate on the printing performance and formation of uniform hydrogel strands (Figure 2C).^[^ ^54^ ^]^ Based on the properties of 30% w/v Pluronic F‐127, simulations have shown that shear‐thinning bioinks with higher viscosities displayed better printing resolution than shear‐thickening inks (thermo‐thickening), thus emphasizing the importance of controlling the bioink viscosity for improved printing resolution.

Strikingly, compared to extrusion bioprinting, drop‐on‐demand bioprinting has very distinct spatial and temporal features. Droplet formation during drop‐on‐demand bioprinting is very fast and complex, and thus difficult to observe the gelation kinetics throughout the experiment (Figure 2D).^[^ ^54^ ^]^ Numerical simulations of droplet formation revealed the importance of the contact angle between the bioink and collecting substrate, which has a distinct surface tension. Therefore, the final shape of each droplet is a function of its surface tension, viscosity, and density, and the droplet‐surface contact angle.

Besides the bioprinting technologies that use printing nozzles (extrusion and drop‐on‐demand), nozzle‐free methods include laser‐induced forward transfer (LIFT)^[^ ^55^ ^]^ and volumetric bioprinting.^[^ ^56^ ^]^ In LIFT, droplets are formed using a laser pulse that focuses on a gold layer adjacent to a donor slide. The laser pulse energy generates a high gas pressure that propels the bioink toward the collector slide. Droplets of bioink formed by LIFT can vary in size (80–140 µm) depending on the laser pulse energy, gap distance, and rheological properties of the bioink. Volumetric bioprinting utilizes multiple visible‐light lasers to solidify the bioink that is contained in a reservoir. Only in defined areas, where the accumulation of several pattern exposures overcomes the gelation threshold, crosslinking will occur. Contrarily to LIFT, volumetric printing requires the use of a photoinitiator that triggers the polymerization upon contact with light. Nozzle‐free bioprinting methods allow for the simultaneous polymerization of entire cross‐sections of the desired 3D shape, rather than sequential printing of bioink droplets or strands. Therefore, such technologies allow for the faster generation of 3D specimens compared to other, classic methods, although only using one type of bioink. Up to now, simulation of bioink polymerization during nozzle‐free printing was not investigated yet but would be very interesting to predict printing resolution.

### 2.3 Modeling the Effect of Bioink Rheology on Shear Stress and Cell Survival

Fluid stresses within the printing nozzle can negatively affect cell survival.^[^ ^30^, ^57^ ^]^ For instance, nozzle shape and size, cell density in the bioink, and bioink viscosity are all parameters that can influence shear stress.^[^ ^28^ ^]^ Therefore, there is a high demand for modeling and simulating shear stress during bioprinting, and its direct impact on cell survival and overall cell activity post‐printing. Shear stress at the printing nozzle can be controlled by varying the printing pressure, bioink viscosity, and nozzle diameter (Figure 2E).^[^ ^30^ ^]^ Shear stress peaked at the wall of the bioink's reservoir and continuously decreased toward the center. A phenomenological model was developed correlating the percentage of live, apoptotic, and necrotic cells with the extrusion bioprinting parameters. Interestingly, this model demonstrated that the dispensing velocity of a non‐Newtonian bioink, as a function of pressure and nozzle diameter, has a high effect on shear stress and cell viability. Experimental validation using cell‐laden alginate bioinks confirmed that increased shear stresses at the printing nozzle were associated with decreased cell viability. For these reasons, in silico simulations should be given higher priority in future studies of bioink‐bioprinting combinations for different biomedical applications.

Computational models of shear stress at the printing nozzle are also relevant for drop‐on‐demand bioprinting.^[^ ^58^ ^]^ Here, the importance of cell localization within each printed droplet was assessed, as shear stresses are differently distributed at off‐center locations inside each droplet.^[^ ^59^ ^]^ Using a Newtonian computational model, which assumes that the spreading of a compact droplet is governed by incompressible Navier–Stokes equations, it was demonstrated that miscellaneous droplet integrity was affected by the stress distribution before and after colliding with a flat surface (printing substrate, Figure 2F). This outcome highlights the need for modeling droplet formation and collision during bioprinting and it can further be utilized to determine appropriate experimental features to preserve post‐printing cell viability.

## 3 Digital Fabrication and Modeling of Fluidic Platforms

The notion of drawing inspiration from nature for the design of novel materials in arts and architecture is not new. Famous architectural constructions by Eiffel, Gaudí, and Paxton, are some examples where nature has been the inspirational source for man‐made creations.^[^ ^60^ ^]^ In the medical field, nature‐inspired materials design for tissue engineering applications is a topic gaining rapid interest among biomedical scientists.^[^ ^60^, ^61^ ^]^ Notwithstanding the exciting advances made in materials design for tissue engineering, the optimization of biological function and natural tissue augmentation, i.e., cell proliferation, ECM production, and tissue growth in vitro, should evolve from a simplistic focus on the materials' physical, mechanical, and chemical properties.^[^ ^62^ ^]^ For these reasons, more complex fluidic platforms are needed for tissue growth and maturation after the fabrication process. New culture systems should be designed to enable multiscale architectural recapitulation of the in vivo tissue, mimic healthy and pathologic events through the optimization of oxygenation and nutrient supply during culture, allow for substance and particle transport from the vascular‐like channel to the tissue in its vicinity, enable native‐like or diseased mechanical stimulation or cues, and provide a systemic physiological performance as observed in vivo.

### 3.1 Design of Fluidic Platforms with Varying Geometry and Size for In Vitro Tissue Culture

**Figure 3:**
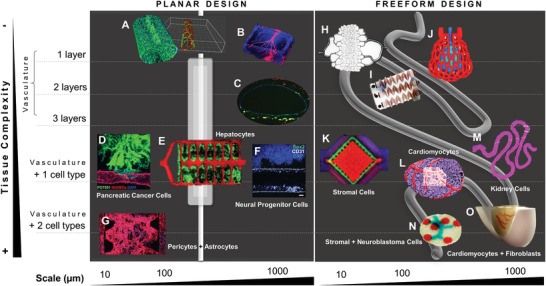
Multiscale planar and freeform designs of fluidic systems used in in vitro tissue culture. A) Microchannel generated in a microfluidic platform and coated with VE‐cadherin‐stained endothelial cells (ECs).^[^ ^68^ ^]^ B) Vascular‐like channel with a diameter of 500 µm printed by 3D micromolding technique with methacrylated gelatin and perfused with ECs.^[^ ^69^ ^]^ C) Vascular‐like channel with a diameter of 1 mm fabricated by drop‐on‐demand bioprinting of ECs (CD31, green), smooth muscle cells (prelabeled DiI, yellow), and fibroblasts (prelabeled DiI, red).^[^ ^70^ ^]^ D) Pancreatic cancer cells invaded toward a biomimetic blood vessel fabricated by micromolding with a diameter of 200 µm.^[^ ^71^ ^]^ E) Hepatocyte aggregates entrapped in a prevascularized vascular network fabricated by projection stereolithography with a channel diameter < 500 µm.^[^ ^72^ ^]^ F) Drop‐on‐demand bioprinted neural progenitor cell‐laden elastin‐like protein bioink in a vascularized tissue‐on‐chip with a diameter of 1 mm.^[^ ^22^ ^]^ G) Coculture of pericytes and astrocytes in an iPSC‐derived EC‐forming network as a blood‐brain‐barrier microvascular model fabricated by soft lithography.^[^ ^73^ ^]^ H) Photolithographic mold for fabrication of a microcirculation model with a channel diameter of 5 µm perfused with blood.^[^ ^79^ ^]^ I) Stenosis microfluidic model fabricated by soft lithography to evaluate platelet adhesion to channel walls with a diameter of 100 µm.^[^ ^80^ ^]^ J) Red blood cell‐perfused air sac with a diameter of 200 µm fabricated by stereolithographic printing of photopolymerizable, food dye photoabsorber hydrogels.^[^ ^72^ ^]^ K) Multimaterial extrusion printed vascularized chip perfused with ECs and cocultured with human mesenchymal stromal cells.^[^ ^74^ ^]^ L) Neonatal‐scale freeform reversible embedding of suspended hydrogels (FRESH) bioprinting of cardiomyocyte‐laden collagen bioinks.^[^ ^75^ ^]^ M) Convoluted renal proximal tubule model fabricated by multimaterial extrusion printing and perfused with ECs and cocultured with kidney cells.^[^ ^77^ ^]^ N) Drop‐on‐demand bioprinted neuroblastoma in vitro model with neuroblastoma cancer cells, stromal cells, and ECs.^[^ ^78^ ^]^ O) Sacrificial writing into functional tissue (SWIFT) fabrication of a perfusable cardiac in vitro tissue with cardiomyocytes, fibroblasts and ECs, and vascular‐like channels with a diameter of about 1 mm.^[^ ^76^ ^]^ Images reproduced and adapted with permission: (A)^[^ ^68^ ^]^ Copyright 2018, the Authors. Published by Springer Nature; (B)^[^ ^69^ ^]^ Copyright 2001, Royal Society; (C)^[^ ^70^ ^]^ Copyright 2018, the Authors. Published by Springer Nature; (D)^[^ ^71^ ^]^ Copyright 2019, the Authors. Published by AAAS; (E)^[^ ^72^ ^]^ Copyright 2019, AAAS; (F)^[^ ^22^ ^]^ Copyright 2020, the Authors. Published by Frontiers; (G)^[^ ^73^ ^]^ Copyright 2019, the Authors. Published by Elsevier; (H)^[^ ^79^ ^]^ Copyright 2001, Royal Society; (I)^[^ ^80^ ^]^ Copyright 2016, the Authors. Published by Springer Nature; (J)^[^ ^72^ ^]^ Copyright 2019, AAAS; (K,L)^[^ ^75^ ^]^ Copyright 2019, the Authors. Published by AAAS; (M)^[^ ^77^ ^]^ Copyright 2016, the Authors. Published by AAAS; (N)^[^ ^78^ ^]^ Copyright 2019, the Authors. Published by MDPI; (O)^[^ ^76^ ^]^ Copyright 2019, the Authors. Published by AAAS.

Over the past decade, multiple examples of fluidic platforms were designed to study specific biological processes and functions underlying human biology. A common feature among all these platforms is the presence, or at least the inclination, to incorporate vascularization in in vitro tissues and models (**Figure** **3**). Vascular networks are the foundation of most mammalian tissues, with some exceptions where oxygenation is scarce, such as in articular cartilage.^[^ ^63^ ^]^ Vascular graft engineering was a pioneering focus in the late 1980s, even before the emergence of the tissue engineering field proposed by Langer and Vacanti.^[^ ^64^, ^65^ ^]^ Back then, the design of artificial blood vessels and vascular grafts was an active research area using synthetic materials compatible with ECs culture. Large diameter (> 5 mm) vascular grafts were successfully developed using polymers, such as polytetrafluoroethylene. However, it was challenging to develop vascular grafts with diameters of less than 5 mm, due to biological reactions at the blood‐ and tissue‐material interfaces that resulted in total occlusion by clotting and scarring. Follow‐up studies used polymers functionalized with cell adhesion ligands (Arg‐Glu‐Asp‐Val) specific for ECs that allowed for a nonthrombogenic response with successful EC adhesion.^[^ ^66^ ^]^ Despite the improved performance of the latter examples, it remains challenging to integrate the best‐engineered vascular grafts with bottom‐up tissue engineering approaches to generate full‐thickness tissues and whole organs. This is partly due to the distinct size of engineered vascular grafts (designed to substitute larger arteries) and the microscale capillaries that are needed to provide nutrients and gas transport to cells.^[^ ^67^ ^]^ In bottom‐up biofabrication, cells are encouraged to self‐assemble and form new capillaries at the micrometric scale (vasculogenesis). Ideally, a combination of bottom‐up and top‐down tissue and organ human‐based replacement approaches may be required for clinical translation where vascular channels inside 3D shapes are pre‐designed and fabricated and enable microvessel sprouting (angiogenesis) to reach all cells in the construct.

As tissue or organ transplantation is severely limited by a critical donor shortage, tissue engineering is yet a discipline that brings hope to restore, maintain, or improve tissue function. Unfortunately, supplying vasculature to in vitro tissues that are thicker than 100–200 µm is a persisting challenge.^[^ ^63^ ^]^ For this reason, incorporating vascular‐like structures in fluidic platforms suitable for in vitro tissue culture is a topic of rising interest. Cardiovascular, neural, and respiratory systems are the most frequently targeted biomedical goals thus far. In disease modeling, tumor models are sought for applications in personalized medicine and drug discovery.

In this section, we divide the design of in vitro culture systems into two major categories: platforms with i) planar and ii) freeform design. Planar models are simpler and easier to fabricate (Figure 3A–G), whereas freeform models are more sophisticated and most frequently generated by automated digital fabrication strategies, such as bioprinting (Figure 3H–O). A planar vascular‐like channel seeded with a monolayer of ECs is the simplest approach (Figure 3A,B).^[^ ^68^, ^69^ ^]^ Bioprinted planar vasculatures with mimicking three‐layered vessels (each layer containing ECs, smooth muscle cells, or fibroblasts) were recently generated (Figure 3C).^[^ ^70^ ^]^ Models with planar vasculatures integrated with one or more cell types, in addition to ECs, were attempted to achieve lumen endothelialization in various studies but did not yet result in native‐like nonleaky blood vessels. For example, a pancreatic cancer in vitro model was developed to assess cancer cell migration toward a biomimetic vessel (Figure 3D).^[^ ^71^ ^]^ Further studies analyzed cocultures of hepatocytes (Figure 3E)^[^ ^72^ ^]^ and neural cells (Figure 3F,G)^[^ ^22^, ^73^ ^]^ with ECs. All these in vitro tissues enabled spatially controlled positioning of vascular cells and tissue‐specific cells as observed in vivo. However, these models are not suitable for functional studies, such as evaluating substance and particle transport from the vascular lumen to the surrounding tissue, and vice‐versa, given the lack of tissue maturation after the fabrication process.

In vitro tissues with enhanced complexity were generated using digital fabrication that allowed for the freeform design of the culture systems. In contrast to planar models, the vasculatures of these systems presented many diversified geometries. Even the simplest platforms that were perfused only with blood cells (Figure 3H–J) displayed exquisite designs and functionalities. A printed lung subunit model composed of an alveolar structure and intricate vascularized topologies was digitally generated by an anisotropic Voroni tessellation, and responded to tidal ventilation and oxygenation upon perfusion with blood (Figure 3J).^[^ ^72^ ^]^ In this model, the oxygen saturation of red blood cells increased with decreasing red blood cell flow rate. This is an encouraging proof of functionality, despite the need for further testing in coculture with respiratory‐specific cells, like respiratory epithelial cells. Another fully printed vascularized model included ECs cocultured with human mesenchymal stromal cells, leading to a dense osteogenic tissue model that enabled the delivery of growth factors transvascularly (Figure 3K).^[^ ^74^ ^]^ This procedure resulted in increased expression of alkaline phosphatase and mineral deposition after 14 d in culture. Therefore, this platform opens new avenues for fabricating in vitro connective tissues, such as bone, fat, and muscle.

In cardiovascular research, two recent studies made striking advances.^[^ ^75^, ^76^ ^]^ In the first study, an organ‐scale tri‐leaflet heart valve was bioprinted with a multiscale vasculature as a neonatal‐scale human heart (Figure 3L).^[^ ^75^ ^]^ This tissue model was fabricated using freeform reversible embedding of suspended hydrogels (FRESH) bioprinting, which supports the shape of fragile cardiomyocyte‐laden collagen bioinks during the 3D assembly process. In FRESH bioprinting, bioinks are dispensed into a slurry bath containing a mixture of gelatin, pluronic F‐127, and gum arabic. This bath has a buoyant effect during the printing process, which is especially advantageous in the generation of 3D structures with complex and freeform topologies. After bioprinting, FRESH was liquefied at 4°C and removed, enabling standard in vitro culture conditions. 14 d after bioprinting, the neonatal‐scale heart model was able to generate electrophysiological spontaneous contractions, demonstrating pinned rotor‐like electrical activity comparable to in vivo. In the second study, a novel bioprinting method was introduced to generate a perfusable cardiac‐like tissue.^[^ ^76^ ^]^ Sacrificial writing into functional tissue (SWIFT) bioprinting was employed to generate in vitro tissues with vascular‐like channels with a diameter of about 1 mm (Figure 3O). Cardiomyocytes and fibroblast spheroids were mixed with a collagen type I/Matrigel solution as organ building blocks and kept at 0°C to 4°C. This ECM‐like slurry bath had a fluid‐like behavior and allowed for 3D‐printing of perfusable vascular channels with a gelatin‐based sacrificial hydrogel. After printing, the 3D constructs were placed at 37 °C enabling simultaneous gelation and stiffening of the slurry bath and removal of the sacrificial hydrogel to leave a vascular network. 8 d after bioprinting, spontaneous cardiomyocyte beating was observed upon the addition of calcium or isoproterenol to the culture medium. In contrast to the FRESH study by Lee and colleagues,^[^ ^75^ ^]^ this novel biomanufacturing method enables a more rapid assembly of perfusable patient‐ and organ‐specific tissues at therapeutic scales. This is possible given the need to print only the vasculature and not the complete 3D construct layer‐by‐layer. However, the printing resolution of the vessels is limited as the ECM‐like slurry contains a high density of cell spheroids, which affects the printing efficiency of the collagen type I/Matrigel solution, and, thus, the stability of the printed structures.

In kidney research, a convoluted renal proximal tubule model was generated by extrusion bioprinting renal cancer cells and sequential seeding with proximal tubule epithelial cells (Figure 3M).^[^ ^77^ ^]^ This model promoted the formation of a tissue‐like epithelium with improved phenotypic and functional properties relative to the same cells grown on 2D controls. Although this model was not able to show its applicability in in vitro drug screening yet, it opens new avenues for creating tissues‐on‐a‐chip as mechanistic drug testing platforms. In another study, an in vitro model of human neuroblastoma was engineered by drop‐on‐demand bioprinting to recapitulate the in vivo microenvironment (Figure 3N).^[^ ^78^ ^]^ Three cell types that belong to the tumor microenvironment were used in this experiment: neuroblastoma cancer cells, stromal cells, and ECs. The vascular‐like channel (blue) was printed with a collagen‐based bioink containing ECs, the stromal support was printed with human mesenchymal stromal cells (green), and the mimicking Homer Wright‐rosettes were printed with neuroblastoma cancer cells (red). This model demonstrated that neuroblastoma cells formed Homer Wright‐like rosettes, maintained their proliferative capacities, and produced a vimentin‐rich matrix. The response of neuroblastoma cancer to drugs can be tested in the future using platforms such as this. Nevertheless, the herein presented neuroblastoma tissue model requires further testing before making its way into clinical precision medicine.

Based on the presented studies in this section, there is an apparent tendency to select freeform over planar manufacturing methods in the future of tissue engineering. Although the use of more sophisticated systems might be more time‐consuming and expensive, due to the use of intricate machinery and prolonged developmental phases, there is a clear preference for designing and fabricating in vitro tissues with increased biological functionality and human relevance.

### 3.2 Computational Modeling of Substance Diffusion and Particle Transport

Shape drives function, as it has become more evident from studies presented in the last sections. Scientists have to reverse engineer the target biological function that tissues should perform in vitro to generate the given tissue at the bench side. With this regard, engineered in vitro tissues are designed to be utilized as drug testing, disease modeling, and/or diagnosis platforms. As such, they should enable substance diffusion and particle transport.

**Figure 4:**
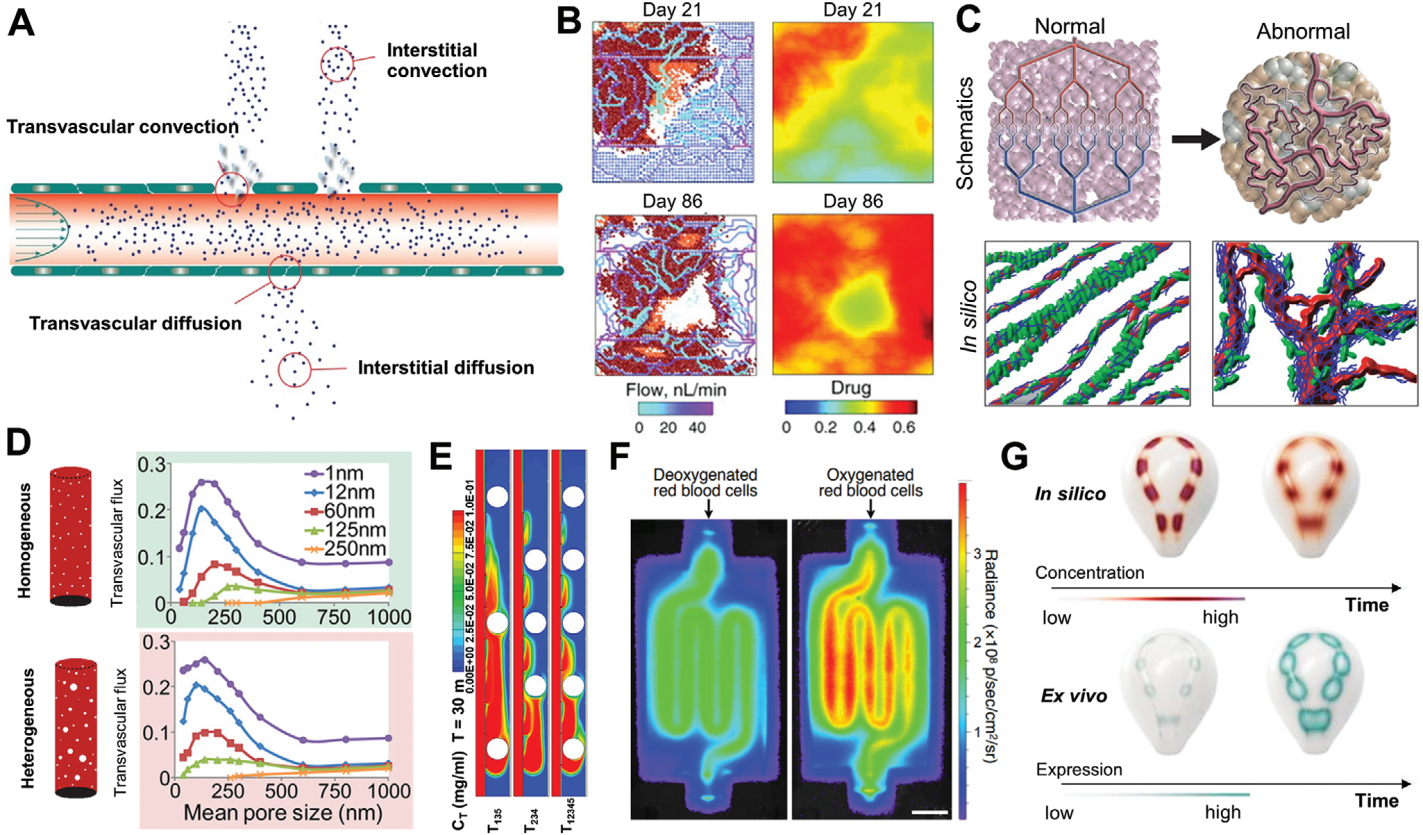
Modeling drug diffusion, particle and gas transport in vivo and in vitro. A) The role of physical forces on the transport of drugs to the target tumor tissue in vivo.^[^ ^83^ ^]^ B) Simulation of drug diffusion in conventional chemotherapy.^[^ ^86^ ^]^ C) Computational modeling of normal and abnormal vasculature in in vivo tumors.^[^ ^87^ ^]^ D) Mathematical simulation of how changes in vascular pore size affect delivery for different sizes of drugs.^[^ ^88^ ^]^ E) Numerical simulation of drug distribution in a tumor‐on‐chip.^[^ ^89^ ^]^ F) Perfused kidney in vitro model expressing firefly luciferase with deoxygenated or oxygenated red blood cells.^[^ ^72^ ^]^ G) Finite modeling of signal diffusion across 3D geometries and experimental dose–response data visualization.^[^ ^7^ ^]^ Images reproduced and adapted with permission: (A)^[^ ^83^ ^]^ Copyright 2018, Elsevier; (B)^[^ ^86^ ^]^ Copyright 2011, AAAS; (C)^[^ ^87^ ^]^ Copyright 2005, AAAS; (D)^[^ ^88^ ^]^ Copyright 2012, Springer Nature; (E)^[^ ^89^ ^]^ Copyright 2019, Elsevier; (F)^[^ ^72^ ^]^ Copyright 2019, AAAS; (G)^[^ ^7^ ^]^ Copyright 2019, Wiley‐VCH GmbH.

Computational modeling plays a key role in predicting substance diffusion and particle transport in vivo.^[^ ^81^, ^82^ ^]^ In cancer research, physical forces, such as transvascular pressure, hydraulic conductivity, and interstitial diffusion in the human vasculature, are used to model in silico the transport of cancer drugs to the target tumor tissue (**Figure** **4**A).^[^ ^83^ ^]^ Also, the shape, size, and elasticity of drug nanocarriers (spherical versus rods) affect intra‐tumor drug distribution, and thus should be considered.^[^ ^84^ ^]^ In neural research, drug development for the treatment of central nervous system diseases is extremely challenging, in large part due to the difficulty in crossing the blood‐brain barrier (BBB).^[^ ^85^ ^]^ Therefore, computational modeling can be a valuable tool to simulate the permeability of the BBB to small molecules, nanoparticles, and nanogels. For these reasons, prediction of drug diffusion and particle transport in engineered in vitro tissues can be modeled in silico before experimental testing.

Hypoxic areas of tumors have poor drug delivery due to their limited vascularization.^[^ ^86^ ^]^ As computational modeling is employed to predict drug diffusion in conventional chemotherapy in vivo (Figure 3B), it would be advantageous to apply in the design of in vitro tissues for visualization of the drug distribution. Other important aspects to be considered in in vitro disease modeling are the shape, size, and permeability of vessel‐like channels. Through the modeling of these parameters in silico, it is possible to mimic the characteristics of abnormal blood vessels in vivo (Figure 3C),^[^ ^87^ ^]^ as well as to spatially and temporally approximate the dynamics of drug delivery in tissues in vitro (Figure 3D).^[^ ^88^ ^]^ For example, a numerical tumor‐on‐chip model was adapted to investigate drug transport (Figure 3E).^[^ ^89^ ^]^ Interestingly, this model demonstrated that drug distribution inside the chip was highly heterogeneous and that tumor size and positioning contribute to this outcome. A lower drug concentration was observed in the tumor channel when they were blocked by larger tumors (> 200 µm). In contrast, the blocking effect of smaller tumors (< 200 µm) was not as significant. This finding was confirmed experimentally using a vascularized tumor‐on‐chip model, indicating a valid accuracy of the numerical simulation. A similar observation was found in a kidney in vitro model perfused with red blood cells (Figure 3F).^[^ ^72^ ^]^ In this case, the red blood cell's ability to deliver oxygen was demonstrated by luminescence, i.e., light emission from oxygenated red blood cells within gels. This study shows that not only nutrient and drug distribution, but also gas transport, are all dependent on the in vitro culture system design.

A new era of biohybrid customizable in vitro tissues and wearables is coming of age.^[^ ^7^ ^]^ Computational models of interaction between a digitally controlled material distribution and biological functionality are the next frontier in biomedical research. Not only have digital fabrication platforms been used to template cells and biomaterials, they were also employed to position exogenous chemical and environmental signals. Such strategies enable cell responses to targeted chemical signals programmed into the printed tissue. In an inspiring study, the release of chemical signals that regulate gene expression in a 3D construct was modeled in silico and later validated with an in vitro model in a repeatable and predictable way (Figure 3G).^[^ ^7^ ^]^ The inducing substance isopropyl *β*‐D‐1‐thiogalactopyranoside (IPTG) was incorporated into acrylic‐based photopolymeric inks (rigid VeroClear (Stratasys RGD810), flexible TangoPlus (Stratasys FLX930), and hygroscopic FullCure Support (Stratasys SUP705)), printed in specific areas, and sprayed with *E. coli*‐loaded agarose hydrogel. *E. coli* cleaved *β*‐galactosidase (an enzyme occurring in *E. coli*) into glucose and galactose (expressed in blue color), demonstrating the metabolic activity of these cells after printing. This novel platform enabled the generation of 3D objects with various protein expression patterns from a single‐input gene circuit using different inducing substances besides IPTG, such as N‐acyl homoserine lactone (AHL) and Rhamnose (RHA). The presented computational model for chemical diffusion dynamics and biological response across arbitrary 3D surfaces might apply to a broad range of applications, including the design and engineering of in vitro tissues.

## 4 Digital Fabrication and Computational Augmented Modeling of In Vitro Tissues

In this section, we focus on biological augmentation strategies used in tissue engineering and how numerical simulation can help to predict the growth of in vitro tissues after the manufacturing process. In order to model and control tissue augmentation in vitro, it is necessary to first master both the macroscopic and microscopic structural output of printed objects. The second challenge is to deconstruct and understand degradation kinetics of the material, cell migration, and cell‐based biomaterial remodeling in 3D. Finally, the last challenge is to predict the natural augmentation, i.e., cell proliferation patterns and amount of ECM production resulting in the growth of biomimetic tissues cultured in artificial platforms.

### 4.1 Design of Macroscopic and Microscopic Input Geometries and Computational Modeling of Output Geometries

**Figure 5:**
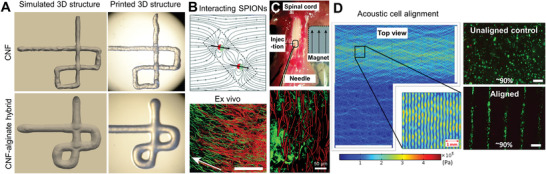
Predicting macroscopic and microscopic 3D structures in in vitro tissues. A) Macroscopic 3D structure of simulated and printed cellulose nanofibril (CNF) and CNF‐alginate hybrid inks.^[^ ^90^ ^]^ B) In silico simulation of anisotropic microgel orientation in the presence of a magnetic field and superparamagnetic iron oxide nanoparticles (SPIONs)^[^ ^93^ ^]^ and in vitro validation of neural cell orientation along the microgels.^[^ ^92^ ^]^ C) In vivo demonstration of nerve cell orientation along magnetically aligned anisotropic microgels.^[^ ^93^ ^]^ D) Computer simulation of osteosarcoma cell alignment in 2% w/v sodium alginate using an acoustic frequency of 2 MHz.^[^ ^94^ ^]^ Viability staining (green) showing 90% of viable cells both in unaligned and aligned samples. Images reproduced and adapted with permission: (A)^[^ ^90^ ^]^ Copyright 2018, the Authors. Published by IOP Publishing; (B)^[^ ^92^ ^]^ Copyright 2017, the Authors. Published by American Chemical Society; (C)^[^ ^93^ ^]^ Copyright 2020, the Authors. Published by The Royal Society of Chemistry; (D)^[^ ^94^ ^]^ Copyright 2019, the Authors. Published by Springer Nature.

Computer‐based simulations are used to predict both the macroscopic targeted 3D structure and the targeted microstructure. Numerical simulation of the macroscopic outcome of two different materials was evaluated and validated experimentally (**Figure** **5**A).^[^ ^90^ ^]^ Although both inks, cellulose nanofibril and cellulose nanofibril‐alginate hybrids, demonstrated varying geometries after extrusion bioprinting, the numerical model was able to predict their shapes very accurate for each material.

Despite the importance of the macroscopic appearance in in vitro tissues, it is the physical and mechanical guidance of cells and gradients of biochemical factors at the microscopic scale that has gained great momentum over the past few years.^[^ ^91^ ^]^ A recent surge of interest in using remote fields, like magnetic, optical, and acoustic, for complex tissue engineering aims at recapitulating the multiscale and hierarchical structure of natural systems. For instance, superparamagnetic nanoparticles were used to induce the alignment of anisometric microgels both in vitro and *inside tissue* for neural cell orientation (Figure 5B,C).^[^ ^92^, ^93^ ^]^ The alignment of the microgels was simulated in silico to support future in vivo experiments (Figure 5B).^[^ ^93^ ^]^ Another study focused on using ultrasound acoustic waves to create uneven pressure fields that can pattern cells into well‐defined geometries (Figure 5D).^[^ ^94^ ^]^ This acoustophoretic method enabled label‐free, i.e., without chemical (chemotaxis) or magnetic (magnetophoresis) labels, patterning of cells encapsulated in 2% w/v alginate hydrogel but the cells did not yet show elongated morphologies after culture. Further optimization of this technology with other biologic and/or fibrous materials may allow for cell elongation and guided cell migration, and ECM remodeling. These studies combined demonstrate that it is crucial to predict material and cell orientation at the microscopic scale for successful tissue remodeling, including in in vitro systems.

### 4.2 Computational Modeling of Cell Migration and Naturally Augmented Tissue Remodeling

**Figure 6:**
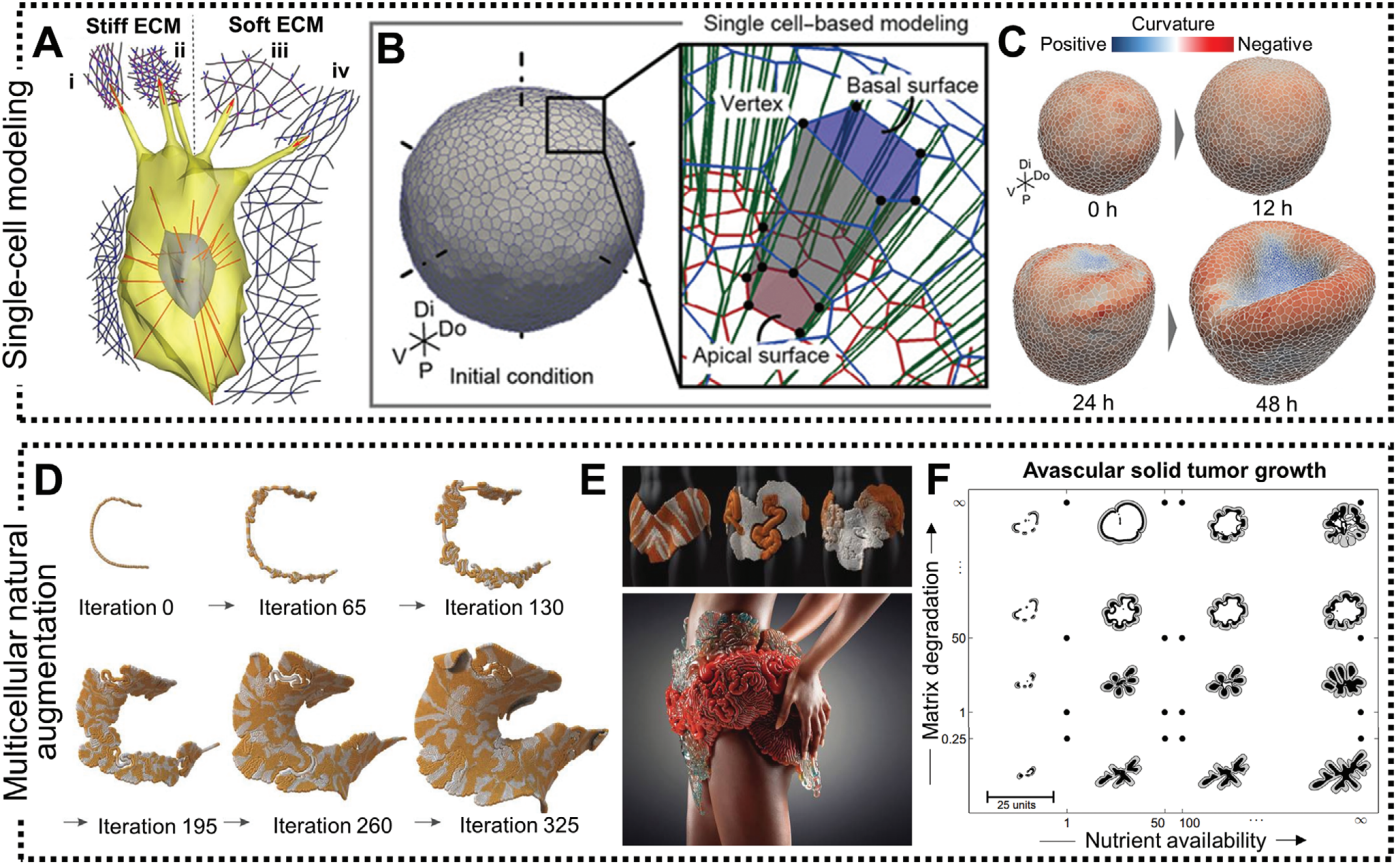
Computational modeling of single‐cell migration and multicellular natural augmentation. A) Single‐cell migration depends on local ECM conditions that dictate the success or failure of traction force generation by focal complexes at the filopodial tips: i) tensioned stiff ECM, ii) relaxed stiff ECM, iii) relaxed soft ECM, and iv) tensioned soft ECM.^[^ ^97^ ^]^ This in silico model was validated in vitro with HUVECs culture in collagen type I hydrogels. B) In silico vertex model of 3D organoid multicellular dynamics when cultured in vitro.^[^ ^99^ ^]^ C) In silico simulation of optic vesicle organoid differentiation into the neural retina (invaginated side) and retinal pigment epithelium (outer cup‐like side).^[^ ^99^ ^]^ D) Continuous computational modeling into the growth of a coherent form by using a mathematical framework applied to a polygonal line. Triangle mesh iterations result in a compact coiled architecture.^[^ ^5^ ^]^ E) Examples of fluidic channel designs generated by the in silico model presented in (D) (up), and a photograph of a 3D printed wearable consisting of a continuous internal network of biocompatible fluidic channels generated by multimaterial additive manufacturing (down).^[^ ^5^ ^]^ F) Multiscale continuum model of avascular solid tumor growth responsive to nutrient availability (horizontal axis) and biomechanical responsiveness (vertical axis).^[^ ^104^ ^]^ Avascular tumor growth kinetics are identical in 3D cancer spheroids cultured in hydrogels in vitro and in the initial growth phase in vivo.^[^ ^104^ ^]^ Images reproduced and adapted with permission: (A)^[^ ^97^ ^]^ Copyright 2018, the Authors. Published by National Academy of Science; (B,C)^[^ ^99^ ^]^ Copyright 2019, the Authors. Published by AAAS; (D,E)^[^ ^5^ ^]^ Copyright 2016, Mary Ann Liebert, Inc.; (F)^[^ ^104^ ^]^ Copyright 2008, Springer Nature.

Cell migration is a multiscale process that integrates signaling, mechanics, and biochemical reaction kinetics.^[^ ^95^ ^]^ Both single and collective movement of cells play an important role in wound healing, cancer invasion, and embryonic morphogenesis.^[^ ^96^ ^]^ Apart from experimental assays that assess cell migration in 3D, it is necessary to develop quantitative computational models to predict and understand the role of matrix mechanics and structure in tissues in vitro and how these parameters regulate cellular migration, growth, and augmentation. In an in silico study, a mathematical model was developed to examine cellular drag force that arises due to resistance to cell motility in 3D environments (**Figure** **6**A).^[^ ^95^ ^]^ This model predicted that at low cell‐matrix adhesiveness values, there is not sufficient traction hence cell velocity is low, whereas, at very high values, the molecular interactions with the matrix are too strong, reducing cell velocity. This means that at low and high adhesiveness values, the model predicts low cell velocity, which was consistent with experimental findings for HUVECs cultured in vitro inside 3D collagen type I gels.^[^ ^97^ ^]^ The drawback of this model is that it only predicts the motility of a single cell, while in vivo and in vitro cell migration most frequently include a population of cells. Collective durotaxis is a key phenomenon occurring in 3D environments that is far more efficient than single‐cell durotaxis. Thus, there is an increasing interest to model collective cell migration in silico.^[^ ^98^ ^]^

Multicellular aggregates like spheroids, organoids, and tissue‐like in vitro systems can be modeled in silico using more sophisticated, continuum growth numerical models.^[^ ^99^ ^]^ The theory of finite kinematic growth was first formalized in the 1990s and rapidly gained popularity with the use of computational models to solve the underlying set of equations.^[^ ^100^ ^]^ This theory requires two additional sets of equations besides the traditional theory of finite elasticity: kinematic and kinetic equations of growth. These two relations have to be prescribed to close the system of governing equations and thus are specific to the type of physiological system—the brain, vasculature, gut, lungs, skin, or heart. The theory of finite growth is based on a particular multiplicative decomposition of the deformation gradient. Physically, this implies that once grown, the initial pieces of a living system can become incompatible and no longer fit together. Thus, these pieces are deformed elastically resulting in growth‐induced residual stresses, which are the hallmark of living tissues that fulfill many functions. Using a relaxation‐expansion 3D vertex model that adequately describes general 3D multicellular dynamics at single‐cell resolution, a recent study perturbed specific cell behaviors, such as cellular contraction, elongation, adhesion, growth, and division, to predict its mechanisms comprehensively (Figure 6B).^[^ ^99^ ^]^ This in silico model deconstructed key cell behaviors, associated with neural retina and retinal pigment epithelium differentiation using a 3D model of the distal part of an optic vesicle (Figure 6C).^[^ ^99^ ^]^

A visionary way of predicting biological augmentation in vitro using continuum computational modeling is possible from the intersection of arts, computational simulation, and biology (Figure 6D).^[^ ^5^ ^]^ This continuum computational model was generated by a mathematical framework applied to a polygonal line. Triangle mesh iterations resulted in diversified coiled architectures, which resembled various biocompatible fluidic channel designs (Figure 6E).^[^ ^5^ ^]^ After simulation, fluidic channels were 3D‐printed using a voxel‐based 3D‐printing technique (inkjet). Droplets of UV‐curable inks (opaque VeroRed (Stratasys), transparent VeroClear (Stratasys), and sacrificial SUP705 and SUP707 (Stratasys)) were deposited layer‐by‐layer in high resolution (600 dpi in *x* and 300 dpi in *y*). For printing hollow channels, sacrificial inks were printed in the lumen of the channels and washed out by high‐pressure water jetting after the printing process was completed. These wearable systems were 3D printed and used as a host environment for the coculture of engineered microorganisms. This study was the first of its kind to demonstrate a simulated continuum growth of 3D printable multimaterial fluidic channels at the product scale. Although the scope of this study was rather focused on the development of wearable hybrid systems, for example as wearable skin that can interact with the human skin as a selective permeable membrane for therapeutic compound delivery, one can potentially utilize the same computational model to simulate the growth of “living” in vitro tissues made of human cells and biomimetic materials.

Finally, one last application of continuum computation is in vitro disease modeling. Instead of using healthy human cells in the manufacturing of tissues in vitro, pathologic cells like cancer cells can be employed to generate 3D replicas of the in vivo pathologic tissue. Hence, modeling tumor growth kinetics with continuum computation has become a topic of increasing interest.^[^ ^101^, ^102^, ^103^ ^]^ Vascular and avascular growth are different phases of growth of solid tumors in vivo.^[^ ^104^ ^]^ The initial avascular phase can be compared to 3D in vitro cultures in the laboratory, whereas the vascular phase involves the process of new blood vessel formation or angiogenesis, which is a core hallmark of cancer. During the avascular phase, cells located in the center of the tumor become deprived of nutrients and give rise to a necrotic core. The outer border of the tumor tissue consists of proliferating cells, and between these two regions is a layer of quiescent or hypoxic cells. Based on this anatomical knowledge, computational simulations were probed to predict how avascular solid tumors grow both in vivo and in vitro (Figure 6F).^[^ ^104^ ^]^ Using this multiscale in silico model, it was demonstrated that solid tumor growth depends on both nutrient availability and biomechanical responsiveness. As divergent nutrient supply and mechanical stimuli affect the morphology and size of grown tumors, it is important to design in vitro culture systems that respond or adapt to these requirements. This could be achieved, for example, through variation of the distance of cancer spheroids to the medium supply, and/or variation of the matrix mechanical properties. The latter can be potentially implemented in in vitro systems by designing adaptable hydrogels, such as living functional hydrogels with bioorthogonal crosslinking that mimic the dynamics of native pathologic conditions.^[^ ^105^ ^]^

## 5 Future Directions

### 5.1 Machine Learning and Serially Linked Naturally Augmented In Vitro Tissues and Organs

Throughout this progress report, we introduced and discussed several studies from the literature that demonstrated how computational modeling can be advantageous when designing and fabricating engineered tissues in vitro. The most likely short‐term application of these “living” systems will be in drug discovery, disease modeling, and personalized medicine. For this reason, digitally fabricated tissues should not only enable architectural replication of native tissues but also be biologically functional. To achieve the required biofunctionality for a given biomedical application, these systems’ fabrication must go through a series of processing and optimization steps. Therefore, we foresee that future research will consider implementing and magnifying digital approaches, such as artificial intelligence and machine learning.^[^ ^106^, ^107^, ^108^ ^]^ Machine learning algorithms can be potentially used in different steps of the fabrication chain. For example, they can be used in patient imaging data retrieval to discover tissue architectures that can be optimized for bioprinting;^[^ ^108^ ^]^ in drug discovery to filter previously tested and/or failed attempts for a given therapy;^[^ ^109^, ^110^ ^]^ in precision medicine to help designing in vitro models with individual genotypic and phenotypic characteristics;^[^ ^111^ ^]^ in material design for optimizing printability requirements;^[^ ^112^ ^]^ and many others. By valuing the intertwining of in silico models and digitalization with the development of in vitro systems, biomedical research will become more human‐relevant compared to state‐of‐the‐art animal models.

Building on our previous projections regarding the use of digital tools in tissue engineering, we are confident that the next frontier in biomedical research is to serially and fluidically link in vitro tissues and organ‐like structures in a systemic approach that mimics human body physiology. In this regard, computational design, digital fabrication, and machine learning will have a major role in deconstructing, simulating, and understanding tissue‐tissue and organ‐organ interplay in vitro.

## Conflict of Interest

The authors declare no conflict of interest.
